# Comparative Analysis of Lifetime Suicide Attempts among Mexican Adolescents, over the Past 12 Years

**DOI:** 10.3390/ijerph18105419

**Published:** 2021-05-19

**Authors:** Rosario Valdez-Santiago, Aremis Villalobos, Luz Arenas-Monreal, Catalina González-Forteza, Alicia Edith Hermosillo-de-la-Torre, Corina Benjet, Fernando A. Wagner

**Affiliations:** 1 CISS, National Institute of Public Health (INSP), Cuernavaca 62100, Mexico; rosario.valdez@insp.mx (R.V.-S.); luz.arenas@insp.mx (L.A.-M.); 2 Department of Epidemiology and Psychosocial Research, National Institute of Psychiatry (INP), Mexico City 14370, Mexico; catiartes@gmail.com (C.G.-F.); cbenjet@gmail.com (C.B.); 3 Psychology Department, Universidad Autónoma de Aguascalientes [Autonomous University of Aguascalientes], Aguascalientes 20131, Mexico; alicia.hermosillo@edu.uaa.mx; 4 School of Social Work, University of Maryland, Baltimore, MD 21201, USA; fernando.wagner@ssw.umaryland.edu

**Keywords:** attempted suicide, adolescence, Mexico

## Abstract

Objective. To compare the occurrence of suicide attempts across nationally representative samples of the Mexican adolescent population over the past 12 years, and to analyze its association with sociodemographic, lifestyle and mental-health indicators. Methodology. Data were drawn from the 2006, 2012 and 2018 National Health and Nutrition Surveys (*n* = 25,056; 21,509; and 17,925 adolescents, respectively). Estimates were based on standardized measurements. Results. The estimated lifetime prevalence rates of suicide attempts were 1.1% in 2006, 2.7% in 2012, and 3.9% in 2018, indicating a 3.4-fold increase. Across the three survey periods, women yielded rates nearly three times higher than men. Lifetime prevalence grew the most among adolescents aged 13–15 years. Compared to the other respondents, the odds of lifetime suicide attempts proved seven times as high for those who had been sexually abused during their childhood, five times as high for those who had been diagnosed with a depressive disorder, three times as high for those who had suffered physical aggression and twice as high for those who had smoked 100+ cigarettes in their lifetimes and those who consumed alcohol. Conclusion: The sharp increase in suicide attempts in Mexico calls for an urgent public-health response via universal and targeted interventions supported by national policy and sustained federal funding.

## 1. Introduction

Suicides are preventable premature deaths. They constitute a global public-health problem which, according to the Global Health Observatory (GHO) of the World Health Organization (WHO) [1], in 2019 accounts for 9.0 deaths per 100,000, adjusted by age and sex. For both sexes, the rates of death by suicide stand at 9.3 in the region of the Americas (Atlas de Salud Mental de la WHO [2018]) and 4.2 in Mexico (Burden of Mental Disorders in the Region of the Americas) [2]. Based on raw and adjusted data, the Mexican rate is lower than the rates reported at the global and regional levels; however, Mexico has yet to reach the 10% reduction objective endorsed by the WHO member states [3].

According to the Global Burden of Disease (GBD) [4], suicide ranks among the five major causes of death for the population between 10 and 24 years old in most regions around the world, and is the third leading cause of death for youth between 15 and 19 years old [5], with similar behavior reported for both sexes. From 2009 to 2014, over 100,000 predominantly male adolescents died by suicide in the Americas: 34,267 were 10–19 and 69,311 15–24 years old. The adjusted mortality rates in that period were 14.9 per 100,000 population in males and 4.0 for women. The adjusted female–male ratio was 3.7 in the Americas region, and the female ratio was highest in Central America, the Hispanic Caribbean and Mexico at 4.4 [6].

A greater understanding of the burden and the determinants of suicide deaths in adolescents is important in order to reduce the problem. According to the Global School-Based Student Health Survey [7], the prevalence of suicide ideation is significantly greater in females than males, in contrast to the prevalence of attempt that did not show differences by sex or age. This study also identified being bullied and not having close friends as factors associated with ideation and attempt.

In Mexico, suicidal behavior is monitored through the National System of Health Surveys, created over 30 years ago. Thus far, more than 20 National Health Surveys have been conducted, salient among them the National Health and Nutrition Surveys (ENSANUTs). Data are also available from the official records provided to the National Institute of Statistics, Geography and Informatics of Mexico (INEGI). According to the INEGI, 7223 individuals took their lives in 2019, 1028 of whom were adolescents 10–19 years old, representing 14.23% of the national total [8]. Based on the 2018 ENSANUT [9], we estimated national prevalence rates of 5.1% and 3.9% for suicidal ideation and attempts among adolescents, respectively. Additionally, the 2016 National Survey on Drug, Alcohol and Tobacco Consumption (ENCODAT) [10] reported a prevalence of 0.5% for last-month suicide attempts (SAs) among 12–17-year-olds.

The 2005 Mexican Adolescent Mental Health Survey (EMSMA) revealed a prevalence of 3.1% for lifetime SAs among adolescents of 12–17 years old in Mexico City (1.6% for men and 4.7% for women), with age 15 representing the period of highest risk [11]. Meanwhile, the prevalence of SAs for last year was 1.09%: 0.57% for men and 1.60% for women [12]. An eight-year follow-up of individuals aged 19 and 23 in 2013 compared with a cohort of the same age and area of residence 12 years earlier found that the prevalence of SAs had increased with moderately significant differences [13]. The National Health Surveys conducted in Mexico over the past two decades provide extensive data on adolescent SA trends.

Some studies in Mexican youth have examined suicide attempt and associated factors in adolescents. A ten-year longitudinal study evaluated annually the association of temperament with suicide ideation in Mexican origin youth beginning at age 12 [7] finding high levels of effortful control and low levels of negative emotionality associated with decreased probability of first onset of suicide ideation, plans, and attempts. A national study conducted in 2007 among a representative sample of students ages 14–19 from public schools, reported the following factors related to suicide attempt: sexual abuse (OR = 1.57), depressive symptoms (OR = 1.51), tobacco use (OR = 2.57), alcohol use (OR = 1.31) and, for women only, having had sexual relations (OR = 1.65) [14]. Consistent with these findings, Dávila and Luna, using data from the National Survey of Student Drug Use (ENCODE, 2012), reported the following factors associated with increased suicide attempts: being female (OR 3.1), under 16 years of age (OR 1.6), suffering a mental illness (OR 3.6), having been forced into sexual contact (OR 2.6); drugs use, smoking or alcohol consumption (OR 1.7, 1.2 and 1.7) [15]. A more recent study with a smaller sample reported similar findings for alcohol, tobacco and marijuana use [16].

In light of the above, we undertook this study in order to compare the occurrence of SAs in the Mexican adolescent population over the past 12 years, and to analyze its association with sociodemographic, lifestyle and mental-health indicators. Analyses were based on three methodologically similar surveys.

## 2. Materials and Methods

We used data from the 2006, 2012 and 2018–2019 waves of the Mexican National Health and Nutrition Survey (ENSANUT). The ENSANUT is a national household study with a probabilistic, multi-stage, stratified and conglomerate sampling design, representative of the population at the national, state and rural/urban levels [17,18,19]. Standardized survey questionnaires target specific age groups: children (0 to 5 years), adolescents (10 to 19 years) and adults (20 and older). Since 2006, those used with adolescents and adults have included items on lifetime SAs; in 2012, the adolescent questionnaire introduced a question on SAs in the previous year.

### 2.1. Sample Selection and Variables of Interest

Our sample consisted of 64,490 adolescents aged 10–19 years: 25,056 in 2006, 21,509 in 2012 and 17,925 in 2018–2019. The household response rate was 87% [18,19].

### 2.2. Lifetime Suicide Attempts

We estimated SA occurrence by posing a direct question: “Have you ever attempted to harm yourself or deliberately injured, cut, intoxicated or hurt yourself in any way with the purpose of dying? According to Remus, “attempters are those who make attempts but fail to end their lives”, and suicide attempt is a type of suicidal behavior defined as: “any action that could cause a person to die” [20]. In 2006 and 2018, the answer options were “Yes, once;” “Yes, two or more times;” and “Never.” In the 2012 survey, the response options to the same question were “Yes, in the last 12 months,” “Yes, but not in the last 12 months” and No, never.” We constructed a dichotomous variable, where 1 = “Yes” and 0 = “No, never.”

For a comparative analysis of the surveys, we identified a set of homogenous demographic, lifestyle and mental-health indicators that were theoretically associated with SA. We analyzed three sets of variables for the three indicators, depending on their availability in the surveys: the demographic variables included sex, age and educational level, categorized as none, elementary school (1–6 years), middle school (7–9 years) and high school or beyond (more than 10 years). They also included type of locality (urban or rural); marital status (not married/nor in union or married/in union); indigenous status (where adolescents spoke an indigenous language); and socioeconomic level. We generated a socioeconomic indicator grouping households into income deciles. To this end, we considered the demographic and socioeconomic characteristics of respondents as well as data from the National Income and Expenditure Survey (ENIGH). This index, which adequately took into account the heterogeneity in the standard socioeconomic variables, was validated by the 2012 ENSANUT and has been described elsewhere [21].

Our lifestyle and mental-health indicators included alcohol and tobacco use, among other variables. Alcohol use was coded as 0 = not current, 1 = occasional and 2 = binge drinking (defined as four drinks for women and five drinks for men on a single occasion, at least once a month). Adolescents who used tobacco were classified into two categories: those who had consumed 100+ cigarettes in their lifetimes and those who had consumed less than that amount or had never consumed tobacco. Also included as lifestyle and mental-health indicators were early sexual initiation, childhood sexual abuse, depressive symptomatology and a clinical depression diagnosis (yes/no), as well as having suffered physical aggression within the past year (yes/no).

The assessment of early sexual initiation included adolescents between the ages of 15 and 19 who initiated their sexual lives before the age of 15, as well as adolescents between the ages of 12 and 14 who initiated their sexual lives before the age of 12. Childhood sexual abuse (yes/no) included adolescents who responded affirmatively to the following open-ended question: “During the course of your life, has anyone fondled or touched you, caressed a part of your body, or had sexual relations with you, when you were very little?” This question was validated nationally.

We measured depressive symptomatology according to the Center for Epidemiological Studies Depression 7 (CESD-7) scale, an abridged seven-item version [22] of the CES-D scale [23] that has been used in Mexican adolescents [24]. Both assess the frequency of depressive symptoms in the week prior to the survey, with nine as the cutoff point for adult respondents suffering from depressive symptomatology. We measured medical diagnosis of depression through the following question: “Have you ever been told by a doctor or another health-care provider that you suffer or have suffered from depression?”

### 2.3. Statistical Analyses

We performed all statistical analyses using STATA SE 14.0 software. Estimates took into account the complex survey design, weighting the data in order to reflect the population of interest and adjusting variance calculations to establish participant clusters as primary sampling units. We computed the frequency of the adolescents’ characteristics according to whether or not they had attempted suicide. We also used chi-squared testing to calculate the statistical significance of the differences in the sociodemographic characteristics of those who had attempted suicide vs. those who had not. We constructed two multivariate regression models to assess SA correlates: (1) the first estimated the likelihood of attempting suicide based on the variables available in the three survey waves (age, sex, tobacco consumption, alcohol abuse, early sexual initiation and having experienced physical aggression within the 12 months prior to the survey); (2) the second model assessed the association between SA and the lifestyle and mental-health indicators in the last 2018–2019 survey wave.

## 3. Results

The lifetime prevalence rates of adolescent SAs in Mexico are shown in Table 1: they rose from 1.1% in 2006 to 2.7% in 2012 and 3.9% in 2018 (at a 95% confidence interval (95% CI)), equivalent to 1.0–1.4, 2.4–3.1, and 3.5–4.3, respectively (Table 1). The largest increase occurred in the 2006–2012 period with a prevalence ratio (PR) of 2.4 (95% CI =1.9–3), in contrast with 1.4 for the 2012–2018 period (95% CI = 1.2–1.7). Lifetime prevalence of adolescent SA grew 3.4 times in the past 12 years (95% CI = 2.8–4.2).

Table 1 also shows our estimates of lifetime SAs by sex. In all three survey periods, the rates for women were nearly three times higher than those of men. For example, in 2006, prevalence was 0.6% (95% CI = 0.4–0.9) for men vs. 1.7% (95% CI = 1.4–2.1) for women. Likewise, the corresponding estimates in the 2018–2019 survey were 1.8% for men vs. 6.1% for women (95% CI = 1.4–2.3 and 5.4–6.7, respectively). These figures reveal a threefold increase for men and a 3.6-fold increase for women between 2006–2018 (95% CI = 2.0–4.6 and 2.9–4.5, respectively.

Finally, Table 1 presents the lifetime prevalence rates and PRs of adolescent SAs by age group and compares them across the surveys analyzed. In the most recent survey, roughly 1% of youth 10–12 years old had attempted suicide at least once in their lifetimes (95% CI = 0.7, 1.3), as opposed to 5.2% of those aged 13–15 (95% CI = 4.4, 6.1), 5.7% of those aged 16–17 (95% CI = 4.8, 6.9) and a comparatively low 4.7% of those aged 18–19 (95% CI = 3.8, 5.6). The last group exhibited the lowest PRs between 2006 and 2018 (2.2, 95% CI = 1.4–3.3). It should be noted that, according to our estimates, the PRs in the 12-year period doubled for this age group, with the highest ratio observed among adolescents aged 13–15 years, for whom prevalence in 2018 was 5.1 times that observed in 2006 (95% CI = 3.7–7.0).

Estimates for SA prevalence in the previous year were available only for the 2012 and 2018–2019 surveys. Overall, 1.4% of youths aged 10–19 indicated last-year SAs in 2012 (95% CI = 1.2–1.7) and 1.8% at least one last-year SA in 2018 (95% CI = 1.6–2.1), with a PR of 1.3% (95% CI = 1.0–1.6) when comparing these estimates to those of the 2006 survey. In 2018, women yielded a higher prevalence of SAs (2.7%; 95% CI = 2.3–3.2) than men (1.0%; 95% CI = 0.7, 1.4).

The age-specific prevalence of SAs in the previous year showed similar increases between 2012 and 2018. The highest rates were observed among adolescents 13–15 and 16–17 years old in both surveys: 2.1% and 2.7% for 13–15-year olds in 2012 and 2018, respectively (95% CI = 1.6–2.6; and 2.2–3.4) and 1.8% and 2.5% for 16–17-year olds, respectively (95% CI = 1.4–2.5; and 1.9–3.4, for 2012 and 2018).

Table 2 includes prevalence estimates for the sociodemographic characteristics of adolescents in every survey. Those with middle- and high-school education yielded the highest lifetime prevalence rates. For example, in the 2018–2019 survey, prevalence stood at 5.7% (95% CI = 5.0–6.6) for middle-school and 4.2% for high-school students (95% CI = 3.5–5.0). The corresponding estimates for 2012 were 3.7% and 3.3%, respectively (95% CI = 3.1–4.4 and 2.5–4.3).

The lifetime prevalence of SAs was higher in all survey periods in urban (1.3%, 3.0%, and 4.1%, with 95% CI = 1.1–1.6; 2.6–3.4; and 3.7–4.6, respectively, for the 2006, 2012, and 2018 surveys), as opposed to rural areas (0.6%, 2.1%, and 3.3%, and 95% CI = 0.4, 0.9; 1.6, 2.7; and 2.8, 4.0, respectively). Those who spoke an indigenous language exhibited a lower lifetime prevalence of SAs (1.2%, 1.0%, and 2.0%, 95% CI = 1.0–1.4; 0.5–2.1; and 1.2–3.1, for survey years 2006, 2012, and 2018, respectively) than those who did not (0.3%, 2.8%, and 4.0%, with 95% CI = 0.1–0.9; 2.5–3.2; and 3.6–4.4, respectively). Finally, the last variable in Table 2 shows higher lifetime prevalence rates of SAs for adolescents in the middle socioeconomic level, as compared to those in the highest and lowest levels. The estimates for 2018 were 4.5%, 3.4%, and 3.8%, respectively, with a 95% CI = 3.8–5.3 for the middle level; 2.9–4.1 for the lowest level; and 3.2–4.6 for the highest level.

Table 3 presents lifetime prevalence of SAs according to risk behaviors and mental-health variables across all surveys. In every survey period, those who had smoked 100+ cigarettes (3.6%, 6.3%, and 10.2% for 2006, 2012, and 2018; 95% CI = 2.5–5.2; 4.8–8.1; 7.8–13.1, respectively) showed higher SA rates than those who had not (0.9%, 2.4%, and 3.6%, with 95% CI = 0.8–1.1; 2.1–2.7; and 3.2–4.0, respectively). Those with higher levels of alcohol use also exhibited a higher lifetime prevalence of SAs. For example, in 2018 the prevalence rate among those who did not use alcohol was 2.5%, compared to 7.1% among those who consumed alcohol infrequently, and 8.2% among those who frequently consumed alcohol (95% CI= 2.2–2.9; 6.1–8.3; and 6.5–10.2, respectively). Similarly, those who had initiated their sexual lives at an earlier age were more likely to have attempted suicide. For example, in 2018, the corresponding prevalence rate among those with an early sexual initiation was 11.7% (95% CI = 8.8–15.3), compared to 7.5% (95% CI = 6.2–9.2) for those who had postponed beginning their sexual lives. 

Table 3 also shows prevalence estimates according to mental-health indicators. In the 2018 survey, adolescents who had suffered childhood sexual abuse showed the highest prevalence rate, at 33.2% (95% CI = 27.2–39.8). The lifetime prevalence rate of SAs among adolescents who had been victims of physical aggression was 14.2% (95% CI = 10.7–18.6), and the prevalence rate for adolescents with depressive symptoms was estimated to be 22.6% (95% CI = 19.5–26.1). Those who had been diagnosed by a physician as having a depressive disorder also exhibited a high prevalence rate of 26.4% (95% CI = 21.8–31.6). 

The primary methods used to attempt suicide among youths 10–19 years old are depicted in Table 4. The most frequently used methods in all survey years were using sharp objects and poisoning with medications (42.4%; 95% CI = 33.8–51.5; 32.3%; 24.6–41.1, respectively, in 2018). However, it should be noted that attempts based on poisoning with medications decreased sharply over the years, from 32.3% (95% CI = 24.6–41.1) in 2006 to 12.2% in 2018 (95% CI = 9.6–15.5), while using sharp objects increased from 42.4% in 2006 (95% CI = 33.8, 51.5) to 79.3% in 2018 (95% CI = 75.3–82.8).

Hospitalization rates associated with SAs also changed over the years. In 2006, an estimated 17.8% of those who had attempted suicide were reported to have been hospitalized (95% CI = 13.0, 23.9), increasing to 19.2% in 2012 (95% CI = 15.0, 24.3), dropping sharply in 2018 to 8.8% (95% CI = 6.6–11.5).

**Figure 1:**
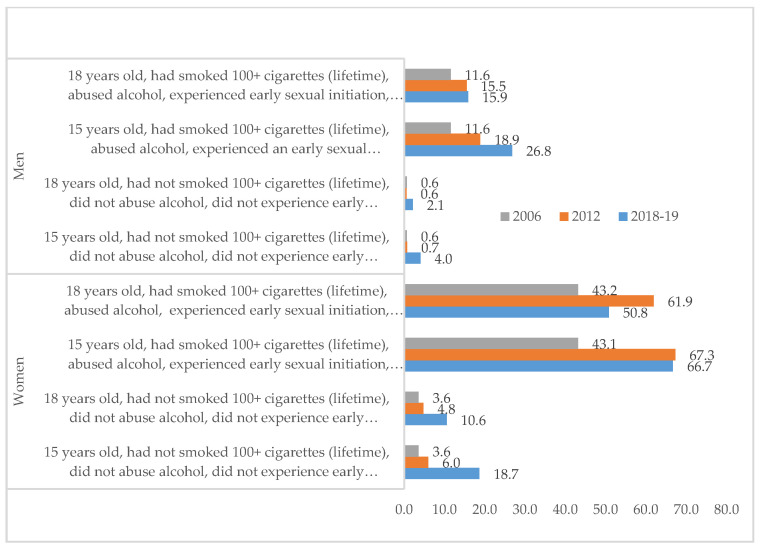
Likelihood (%) of lifetime suicide attempts in adolescents (10–19 years old) according to different population profiles. Mexican National Health and Nutrition Surveys 2006, 2012, & 2018–2019.

Figure 1 displays the likelihood of attempting suicide over the course of a lifetime broken down by sex and factoring in tobacco consumption, alcohol abuse, early sexual initiation and having been the victim of violence in the 12 months prior to the survey. Figure 1 depicts a complex pattern of association, highlighting the following features: (a) in almost every category, women were more likely to attempt suicide than men; (b) regardless of sex, those with histories of smoking, binge drinking, early sexual initiation and having been the victims of violence were most likely to attempt suicide; (c) for both men and women, those 15 years old were more likely to have attempted suicide than those 18 years old; and, finally, (d) the probability of SAs increased over time for all but two groups (spikes occurred among women 15 and 18 years old in the 2012 survey).

Table 5 presents results from our logistic regression analysis of the association between SAs and lifestyle and mental-health indicators using data from the latest survey. Women were almost three times as likely as men to attempt suicide during their lifetimes (OR = 2.82, 95% CI = 1.84, 4.32). Adolescents who had smoked 100+ cigarettes during their lifetimes (OR = 1.92; 95% CI = 1.07, 3.44) were almost twice as likely during their lifetime to attempt suicide than those who had not. Those who had consumed alcohol were almost twice as likely to attempt suicide during their lifetimes than those who had not (OR = 1.97; 95% CI = 1.27, 3.06 for those with infrequent use and OR = 2.23; 95% CI = 1.28, 3.88 for those with frequent use). Adolescents who had suffered childhood sexual abuse were seven times more likely to attempt suicide those who had not (OR = 7.15, 95% CI = 4.21, 12.3). Adolescents diagnosed by a physician as having a depressive disorder were five times more likely to attempt suicide during their lifetimes (OR = 5.18, 95% CI = 3.32, 8.07), while victims of physical aggression were three times more likely to make such an attempt during their lives compared to those who had not been victimized (OR = 3.15, 95% CI = 1.87, 5.29).

## 4. Discussion

### 4.1. Noteworthy Findings

By comparing three waves of the National Health and Nutrition Surveys conducted over 12 years, we aimed to assess changes in the rate of suicide attempts among adolescents and identify their sociodemographic, lifestyle and mental-health correlates. Our study yielded four significant findings: first, the prevalence of lifetime SAs among adolescents in Mexico rose more than threefold in slightly over a decade, with a particularly steep increase occurring between 2006 and 2012. This is consistent with findings from studies of somewhat older young adults in the Mexico City area that found a twofold increase (marginally significant) for SAs from 2001 to 2013 [25].

A second notable finding is that SAs increased sharply while SA-related hospitalizations decreased over time, from 17.8% in 2006 to 8.8% in 2018. It is unclear, however, whether this is a result of changes in the methods used to attempt suicide. We found a decrease in attempts using poison (medications, narcotics, alcohol, solvents, chemicals and pesticides), but an increase in the use of sharp objects during the period studied. It is possible that people attempting suicide by means of poisoning were more promptly taken or referred to hospitals during this period, while those who cut themselves were treated at home or in other health-care facilities. Our survey did not assess the severity of the attempts, which is likely to play an important role in whether or not an adolescent is taken to a hospital or other facility following a SA.

Third, while overall women suffered from a SA rate three times higher than that of men, the increase in the prevalence of attempts was similar for both sexes, although slightly lower for men. As would be expected, older adolescents showed a greater lifetime occurrence of SAs. However, the sharpest increase over time was observed in the 13-to-15-year-old age group, with a fivefold increase in slightly over a decade. This suggests that younger generations are at increasing risk for attempting suicide, and portends greater challenges for public health in the future.

Fourth, we identified numerous SA correlates. In the most recent cohort, we found the highest prevalence of SAs among the following groups: women, non-indigenous youth, adolescents 13–15 years old, and those who lived in urban areas, belonged to the middle socioeconomic class, consumed more alcohol, had smoked 100+ cigarettes in their lifetimes, had experienced an early sexual initiation, had been sexually abused during childhood, had suffered a physical aggression, had a diagnosis of depression and displayed depressive symptoms. Many of these correlates have previously been reported in international contexts and have also been observed among Mexican adolescents specifically [26,27,28].

### 4.2. These Findings in the Context of Mexico

These results raise an important question: why did adolescent SAs in Mexico increase so sharply from 2006 to 2018? The adolescents who responded to the surveys in 2006, 2012 and 2018 were born after the 1980s. In Mexico, the government began to implement neoliberal policies at that time, aimed at reducing social spending. This carried a number of consequences, among them a decline in sources of employment in the formal sector accompanied by an increase in poverty and social inequality [29]. In 2018, 41.9% of the Mexican population lived in poverty, with 7.4% living in extreme poverty [30]. Against this backdrop, the majority of adolescents have been caught up in a vicious cycle of deprivation, finding it difficult to continue their education or obtain employment, and leaving them with discouraging future prospects.

In addition, Mexico has seen a sharp increase in violence stemming from the war on drugs and drug cartels, particularly during the six-year presidential term from 2006–2012, negatively influencing various indicators of violence. In fact, drug cartels have expanded their presence and scope, becoming involved in a variety of violent and illegal activities, such as theft, kidnapping and assaults. Young people are recruited or forced to work for the cartels, exposing them to otherwise unimaginable trauma. However, there is no escaping these organizations once ensnared in their webs, perhaps pushing some young people to seek death by suicide or by being involved in homicide. The intentional homicide rate in the country rose from 9.7 per 100,000 inhabitants at the beginning of the presidential term in 2006 [31] to 17.9 in 2010, reversing the decreasing tendency of the two previous decades. This increase in social violence has affected mental health. A study conducted in Mexico among students in their final years of medical school found that those living in the most violent areas had a significantly higher risk of anxiety, depression and suicidal ideation [32].

At the same time, drug use has increased significantly in Mexico in recent decades, particularly among youth. Patterns of drug use have become more complex, posing new challenges for an already overburdened mental-health system. These nationwide problems have become even more severe given the lack of a clearly defined national policy, legislation and adequate resources devoted to mental health in general and suicide prevention in particular [33].

### 4.3. Limitations

This study has a number of strengths including a large representative sample, robust sampling methods and high comparability across surveys. However, we should note several limitations. The cross-sectional survey design does not provide evidence for causality or even indicate the directionality of the associations between SAs and their sociodemographic, lifestyle and mental-health correlates. For example, while high levels of alcohol use might be a contributing factor in attempted suicide (i.e., by reducing inhibitions) [34,35], SAs might lead to increased alcohol consumption as a method of self-medication. It is also possible other variables account for this association (poverty, sexual abuse and prior mental disorders such as depression, among others) [36].

Another limitation is that measuring SAs depends on self-reporting regarding a sensitive issue with cultural and religious taboos. Hence, our prevalence estimates are likely to be conservative. In fact, a study of Mexican adolescents found that adolescents were 48% less likely to self-report a SA when an adult (usually a parent) was present during the household survey [32]. Despite these limitations, our study makes an important contribution to the discussion of public policy and suicide prevention.

The increase in SAs among Mexican adolescents over the decade in question underscores the need for long-term and scalable actions. Maintaining political will and implementing public policies require national policy enacted into legislation and supported by adequate financial resources. There is no national law regarding mental health or suicide prevention in Mexico. A recent review of mental-health and suicide-specific legislation in the country found that only 14 of 32 states have enacted mental-health legislation, and only two states suicide-specific legislation [37].

Legislation addressing mental health concerns and aiming to prevent SAs and death from suicide should include specific policy proposals and allocate adequate financial resources. The wide range of SA correlates that this and other studies have found suggests a heterogeneity of high-risk groups [37]. This implies both equifinality (a diversity of pathways leading to the same outcome, i.e., a SA) and multi-finality (the same risk factors, e.g., depressive symptoms and childhood sexual abuse, leading to divergent outcomes) [38]. This highlights the need for both universal and targeted interventions.

Our results also underline the importance of early intervention, as younger age groups are increasingly at risk. For those in late childhood as well as adolescents, school interventions (particularly in middle school) are ideal given that schools enjoy easier and more universal access to this population. This positions them to monitor the increase in the frequency of these behaviors during this crucial period. It is also essential to incorporate relevant stakeholders such as educators and parents. In addition, given the magnitude of the problem, community-level interventions are likely to have a greater impact on public health than individual-level interventions based in the health sector. Effective strategies might include working with the media and engaging popular teenage artists –and the entertainment industry more generally—to promote specific messaging and preventive strategies through targeted therapeutic games and recreational activities [39].

Finally, it is important to note that the most recent survey, conducted in 2018, took place before the onset of the COVID pandemic. Many have observed the likely deleterious consequences of the COVID-19 pandemic on mental health [40]. Although initial data on suicide trends during the early months of the pandemic across 21 countries including Mexico found no evidence of an increase in suicide deaths, this study did not examine rates in adolescents specifically and only included the early months of the pandemic [40]. So we may expect SAs to continue to rise even more sharply in Mexico in the future. Public policy and actions are therefore urgently needed to address the widening sense of despair among young people.

## 5. Conclusions

The sharp increase in suicide attempts in Mexico calls for an urgent public-health response via universal and targeted interventions supported by national policy and sustained federal funding.

## Figures and Tables

**Table 1:** Prevalence ratios and 95% confidence intervals for lifetime suicide attempts in adolescents (10–19 years old) by sex and age group. Mexican National Health and Nutrition Surveys 2006, 2012 and 2018–2019.

	2006	2012	2018–2019	2006 vs. 2012	2012 vs. 2018	2006 vs. 2018–2019
	% [95% CI]	% [95% CI]	% [95% CI]	PR [95% CI]	PR [95% CI]	PR [95% CI]
Lifetime suicide attempt (s)	1.1 [1.0,1.4]	2.7 [2.4,3.1]	3.9 [3.5,4.3]	2.4 *** [1.9, 3.0]	1.4 *** [1.2,1.7]	3.4 *** [2.8,4.2]
Sex						
Men	0.6 [0.4,0.9]	0.9 [0.6,1.2]	1.8 [1.4,2.3]	1.4 [0.9,2.3]	2.1 *** [1.4,3.1]	3.0 *** [2.0,4.6]
Women	1.7 [1.4,2.1]	4.6 [4.0,5.3]	6.1 [5.4,6.7]	2.7 *** [2.1,3.5]	1.3 ** [1.1,1.6]	3.6 *** [2.9,4.5]
Age Group						
10–12 years	0.4 [0.2,0.5]	1.1 [0.8,1.5]	1.0 [0.7,1.3]	3.0 *** [1.8,5.0]	0.9 [0.6,1.4]	2.8 *** [1.7,4.5]
13–15 years	1.0 [0.8,1.3]	3.5 [2.8,4.2]	5.2 [4.4,6.1]	3.4 *** [2.4,4.8]	1.5 ** [1.2,2.0]	5.1 *** [3.7,7.0]
16–17 years	1.8 [1.3,2.3]	3.8 [3.0,4.8]	5.7 [4.8,6.9]	2.2 *** [1.5,3.1]	1.5 ** [1.1,2.0]	3.3 *** [2.3,4.6]
18–19 years	2.1 [1.5,3.1]	3.2 [2.4,4.4]	4.7 [3.8,5.6]	1.5 [0.9,2.5]	1.4 * [1.0,2.1]	2.2 *** [1.4,3.3]
Last-year suicide attempt(s)	NA	1.4 [1.2,1.7]	1.8 [1.6,2.1]	NA	1.3 * [1.0,1.6]	NA
Sex						
Men	NA	0.5 [0.4,0.8]	1.0 [0.7,1.4]	NA	1.8 * [1.1,3.0]	NA
Women	NA	2.4 [2.0,2.9]	2.7 [2.3,3.2]	NA	1.1 [0.9,1.5]	NA
Age Group						NA
10–12 years	NA	0.7 [0.5,1.1]	0.7 [0.5,1.0]	NA	0.9 [0.6,1.7]	NA
13–15 years	NA	2.1 [1.6,2.6]	2.7 [2.2,3.4]	NA	1.3 [0.9,1.9]	NA
16–17 years	NA	1.8 [1.4,2.5]	2.5 [1.9,3.4]	NA	1.4 [0.9,2.1]	NA
18–19 years	NA	1.3 [0.8,2.2]	1.4 [1.0,2.0]	NA	1.1 [0.6,2.1]	NA

Notes: sample sizes (25,056 for 2006, 21,509 for 2012 and 17,925 for 2018–2019). Represented population in thousands (22,875 for 2006; 22,804.1 for 2012; and 22,886 for 2018–2019). Weighted estimates; * *p* < 0.05 ** *p* < 0.01 *** *p* < 0.001, CI = Confidence interval, NA = Not available.

**Table 2:** Prevalence of lifetime suicide attempts in adolescents (10–19 years old) by sociodemographic characteristics and survey year. Mexican National Health and Nutrition Surveys 2006, 2012, 2018–2019.

	2006		2012		2018–2019	
	%	(95% CI)	%	(95% CI)	%	(95% CI)
Educational level						
None	1.4 ***	(0.4,4.6)	4.0 ***	(0.9,15.6)	0.2 ***	(0.0,1.6)
Elementary	0.6	(0.5,0.8)	1.5	(1.2,2.0)	1.7	(1.3,2.2)
Middle	1.4	(1.0,1.9)	3.7	(3.1,4.4)	5.7	(5.0,6.6)
High school or beyond	1.8	(1.3,2.3)	3.3	(2.5,4.3)	4.2	(3.5,5.0)
Area of residence						
Urban	1.3 **	(1.1,1.6)	3.0 *	(2.6,3.4)	4.1 *	(3.7,4.6)
Rural	0.6	(0.4,0.9)	2.1	(1.6,2.7)	3.3	(2.8,4.0)
Marital Status ^&,^**						
Not married/in union	1.3	(1.1,1.6)	3.1	(2.7,3.6)	3.6 **	(3.3,4.0)
Married/in union	2.2	(1.4,3.6)	5.7	(3.7,8.8)	9.7	(7.1,13.2)
Spoke an indigenous language						
No	0.3 **	(0.1,0.9)	2.8 **	(2.5,3.2)	4.0 **	(3.6,4.4)
Yes	1.2	(1.0,1.4)	1.0	(0.5,2.1)	2.0	(1.2,3.1)
Socioeconomic level						
Low	1.1	(0.8,1.3)	2.5	(2.1,3.0)	3.4	(2.9,4.1)
Middle	1.3	(1.0,1.8)	3.3	(2.5,4.2)	4.5	(3.8,5.3)
High	1.5	(1.0,2.3)	2.8	(2.2,3.7)	3.8	(3.2,4.6)

Notes: sample sizes (25,056 for 2006, 21,509 for 2012 and 17,925 for 2018–2019). Represented population in thousands (22,875 for 2006; 22,804.1 for 2012; and 22,886 for 2018–2019), Weighted estimates; * *p* < 0.05 ** *p* < 0.01 *** *p* < 0.001, CI = Confidence interval, ^&^ = includes adolescents aged 12 years or more.

**Table 3:** Prevalence of lifetime suicide attempts in adolescents (10–19 years old) by lifestyle and mental-health indicators, Mexican National Health and Nutrition Surveys 2006–2018–2019.

	2006		2012		2018–2019	
	%	(95% CI)	%	(95% CI)	%	(95% CI)
Total	1.1	(1.0,1.4)	2.7	(2.4,3.1)	3.9	(3.5,4.3)
Lifestyle indicators						
100+ cigarettes (lifetime)						
No	0.9 **	(0.8,1.1)	2.4 **	(2.1,2.7)	3.6 **	(3.2,4.0)
Yes	3.6	(2.5,5.2)	6.3	(4.8,8.1)	10.2	(7.8,13.1)
Alcohol use						
None	0.7 ***	(0.6,0.9)	1.3 ***	(1.0,1.7)	2.5 ***	(2.2,2.9)
Infrequent	2.2	(1.6,3.0)	4.4	(3.7,5.2)	7.1	(6.1,8.3)
Frequent	4.8	(3.3,6.8)	5.6	(4.0,7.7)	8.2	(6.5,10.2)
Early sexual initiation ^&,^**						
No	2.9	(1.9,4.3)	3.8 *	(2.9,5.1)	7.5 *	(6.2,9.2)
Yes	5.3	(3.2,8.7)	9.0	(5.9,13.5)	11.7	(8.8,15.3)
Mental-health indicators						
Childhood sexual abuse **						
No	NA	-	NA	-	3.2 ***	(2.8,3.5)
Yes	NA	-	NA	-	33.2	(27.2,39.8)
Victim of physical aggression (last year)						
No	1.1 *	(0.9,1.3)	2.4 ***	(2.1,2.7)	3.5 ***	(3.1,3.9)
Yes	4.1	(2.5,6.7)	11.9	(8.7,16.1)	14.2	(10.7,18.6)
Depressive symptoms (last week) **						
No	NA	-	NA	-	2.7 ***	(2.4,3.1)
Yes	NA	-	NA	-	22.6	(19.5,26.1)
Depressive disorder diagnosed by a physician **						
No	NA	-	NA	-	3.0	(2.7,3.4)
Yes	NA	-	NA	-	26.4 ***	(21.8,31.6)

Notes: sample sizes (25,056 for 2006, 21,509 for 2012 and 17,925 for 2018–2019). Represented population in thousands (22,875 for 2006; 22,804.1 for 2012; and 22,886 for 2018–2019), Weighted estimates; * *p* < 0.05 ** *p* < 0.01 *** *p* < 0.001, CI = Confidence interval, NA = Not available, ^&^ = includes adolescents aged 12 years or more.

**Table 4:** Characteristics of lifetime suicide attempts in adolescents (10–19 years old), Mexican National Health and Nutrition Surveys 2006, 2012, & 2018–2019.

	2006		2012		2018–2019	
	%	CI	%	CI	%	CI
Primary method						
Poisoning with medications	32.3	(24.6,41.1)	25.8	(20.4,32.0)	12.2	(9.6,15.5)
Poisoning with narcotics	1.8	(0.8,4.1)	0.8	(0.4,1.7)	0.2	(0.1,0.6)
Poisoning with alcohol	2.2	(0.9,5.0)	1.4	(0.6,2.9)	0.5	(0.2,1.3)
Poisoning by inhaling organic hydrocarbon solvents	1.1	(0.2,7.5)	0	-	0.5	(0.1,2.4)
Poisoning with pesticides	1.3	(0.5,3.4)	0.7	(0.3,1.9)	0.6	(0.1,2.5)
Poisoning with chemical products (e.g., acids, anti-corrosives)	2.3	(0.8,6.1)	0.3	(0.1,0.6)	0.5	(0.2,1.2)
Strangling	5.7	(3.0,10.6)	5.1	(3.1,8.2)	3.5	(2.2,5.5)
Guns and other arm weapons	1.6	(0.3,7.2)	0.5	(0.1,3.1)	0.5	(0.1,2.0)
Burning	7.9	(3.5,17.0)	0.4	(0.1,1.3)	0.8	(0.3,2.0)
Sharp objects	42.4	(33.8,51.5)	59.7	(52.9,66.2)	79.3	(75.3,82.8)
Jumping from height or in front of a moving vehicle	2	(1.1,3.6)	0.6	(0.2,1.7)	1.3	(0.6,2.8)
Other	6.6	(3.5,12.1)	6.8	(3.6,12.5)	3.7	(2.3,6.0)
Did not answer			0.4	(0.1,1.4)	2.7	(1.6,4.4)
Hospitalization						
Yes	17.8	(13.0,23.9)	19.2	(15.0,24.3)	8.8	(6.6,11.5)
No	82.2	(76.1,87.0)	80.8	(75.7,85.0)	91.2	(88.5,93.4)

**Table 5:** Results of multivariate logistic regression analysis of lifetime suicide attempts in adolescents (10–19 years old). Mexican National Health and Nutrition Survey 2018–2019.

	OR	*p* Value	[95% CI]
Sex			
Men	1.00	referent	
Women	2.82	<0.001	(1.84, 4.32)
Age group			
10–12 years	1.00	referent	
13–15 years	2.64	<0.001	(1.69, 4.13)
16–17 years	1.47	0.196	(0.82, 2.63)
18–19 years	0.61	0.105	(0.34, 1.11)
100+ cigarettes (lifetime)			
No	1.00	referent	
Yes	1.92	0.028	(1.07, 3.44)
Alcohol use			
None	1.00	referent	
Infrequent	1.97	0.002	(1.27, 3.06)
Frequent	2.23	0.004	(1.28, 3.88)
Childhood sexual abuse			
No		referent	
Yes	7.15	<0.001	(4.21, 12.13)
Depression diagnosed by a physician			
No		referent	
Yes	5.18	<0.001	(3.32, 8.07)
Victim of physical aggression (last year)			
No	1.00	referent	
Yes	3.15	<0.001	(1.87, 5.29)

Notes: sample size = 17,925 adolescents; represented population in thousands (22,886), F adjusted = 0.228, OR = Odds Ratio, CI = Confidence interval.

## Data Availability

The public use of ENSANUT data is archived at the National Institute of Public Health, Mexico.

## References

[1] Global Health Observatory SDG Target 3.4|Noncommunicable Diseases and Mental Health Data by Country. https://apps.who.int/gho/data/view.main.SDG34v?lang=en.

[2] Pan-American Health Organization (2018). The Burden of Mental Disorders in the Region of the Americas.

[3] World Health Organization (2014). Preventing Suicide: A Global Imperative.

[4] Naghvi M. (2019). Global, regional and national burden of suicide mortality 1990 to 2016: Systematic analysis for the Global Burden of Disease Study. BMJ.

[5] World Health Organization Home Page. https://www.who.int/es/news-room/fact-sheets/detail/suicide.

[6] Panamerican Health Organization (2021). Suicide Mortality in the Americas. Regional Report 2010–2014.

[7] Lawson K.M., Kellerman J.K., Kleiman E.M., Bleidorn W., Hopwood C.J., Robins R.W. The role of temperament in the onset of suicidal ideation and behaviors across adolescence: Findings from a 10-year longitudinal study of Mexican-origin youth. J. Personal. Soc. Psychol..

[8] Instituto Nacional de Estadística Geografía e Informática Defunciones por Suicidio por Entidad Federativa y Grupos de Edad Según Sexo 2010–2019. https://www.inegi.org.mx/app/tabulados/interactivos/?pxq=Salud_Mental_06_0ce246dc-784f-4198-807b-4375a1612693.

[9] Rivera-Rivera L., Fonseca-Pedrero E., Séris-Martínez M. (2020). Prevalencia y factores psicológicos asociados con conducta suicida en adolescentes ENSANUT 2018–19. Salud Pública Mex..

[10] Villatoro Velázquez J.A., Reséndiz Escobar E., Mujica Salazar A., Instituto Nacional de Psiquiatría Ramón de la Fuente Muñiz (2017). Encuesta Nacional de Consumo de Drogas, Alcohol y Tabaco 2016–2017: Reporte de Alcohol.

[11] Borges G., Benjet C., Medina-Mora M.E., Orozco R., Nock M. (2008). Suicide ideation, plan, and attempt in the Mexican adolescent mental health survey. J. Am. Acad. Child Adolesc. Psychiatry.

[12] Borges G., Orozco R., Benjet C., Medina-Mora M.E. (2010). Suicidio y conductas suicidas en México: Retrospectiva y situación actual. Salud Pública Mex..

[13] Borges G., Benjet C., Orozco R., Medina-Mora M.E. (2017). The growth of suicide ideation, plan and attempt among young adults in the Mexico City metropolitan area. Epidemiol. Psychiatr. Sci..

[14] Pérez-Amezcua B., Rivera-Rivera L., Atienzo E.E., De Castro F., Leyva-López A., Chávez-Ayala R. (2010). Prevalencia y Factores Asociados a la Ideación e Intento Suicida en Adolescentes de Educación Media Superior de la República Mexicana. Salud Publica Mex..

[15] Dávila-Cervantes C.A., Luna-Contreras M. (2019). Suicide attempt in teenagers: Associated factors. Rev. Chil. Pediatr..

[16] Arenas-Monreal L., Hidalgo-Solórzano E., Chong-Escudero X., Durán-De la Cruz J.A., González-Cruz N., Pérez-Matus S., Valdez-Santiago R. (2021). Suicidal behaviour in adolescents: Educational Interventions in Mexico. Health Soc. Care Community.

[17] Shamah-Levy T., Villalpando-Hernández S., Rivera-Domarco J.A. (2007). Resultados de Nutrición de la ENSANUT 2006.

[18] Gutierrez J.P., Dommarco J., Shamah-Levy T., Villalpando-Hernandez S., Franco A., Cuevas-Nasu L., Romero-Martinez M. (2012). Encuesta Nacional de Salud y Nutrición 2012. Resultados Nacionales.

[19] Romero-Martínez M., Shamah-Levy T., Vielma-Orozco E., Heredia-Hernández O., Mojica-Cuevas J., Cuevas-Nasu L., Rivera-Domarco J. (2019). Encuesta Nacional de Salud y Nutrición 2018–2019: Metodología y perspectivas. Salud Pública Mex..

[20] Romero-Martínez M., Shamah-Levy T., Franco-Nuéz A., Villalpando S., Cuevas-Nasu L., Gutiérrez J.P., Rivera-Dommarco J.A. (2013). Encuesta Nacional de Salud y Nutrición 2012: Diseño y cobertura. Salud Pública Mex..

[21] Remus R. (2020). Suicide in Adolescence: A Review of Literature. Rev. Asistenta Soc..

[22] Gutiérrez J.P. (2013). Clasificación socioeconómica de los hogares en la ENSANUT 2012. Salud Pública Mex..

[23] Salinas-Rodríguez A., Manrique-Espinoza B., Acosta-Castillo I., Téllez-Rojo M., Franco-Núñez A., Gutiérrez-Robledo L., Sosa-Ortiz A. (2013). Validación de un punto de corte para la Escala de Depresión del Centro de Estudios Epidemiológicos, versión abreviada (CESD-7). Salud Publica Mex..

[24] Radloff L. (1977). The CES-D Scale: A Self-Report Depression Scale for Research in the General Population. Appl. Psychol. Meas..

[25] González-Forteza C., Solís C., Jiménez A., Hernández I., González-González A., Juárez F., Medina-Mora M.E., Fernández Varela H. (2011). Confiabilidad y validez de la escala de depresión CES-D en un censo de estudiantes de nivel medio superior y superior, en la Ciudad de México. Salud Ment..

[26] Hermosillo-de la Torre A.E., González-Forteza C., Rivera-Heredia M.E., Méndez-Sánchez C., González-Betanzos F., Palacios-Salas P., Jiménez A., Wagner F.A. (2020). Understanding suicidal behavior and its prevention among youth and young adults in Mexico. Prev. Med..

[27] Campisi S.C., Carducci B., Akseer N., Zasowski C., Szatmari P., Bhutta Z.A. (2020). Suicidal behaviours among adolescents from 90 countries: A pooled analysis of the global school-based student health survey. BMC Public Health.

[28] Tello C. (2012). Sobre la Desigualdad en México.

[29] Consejo Nacional de Evaluación (2019). Dirección de Información y Comunicación Social. 10 Años de Medición de Poblreza en México, Avances y Retos en Política Social.

[30] Escalante G.F. (2009). El Homicidio en México Entre 1990 y 2007: Aproximación Estadística.

[31] Escobar-Padilla B., Márquez-González H., Consejo y Chapela C., López-Sepúlveda A.C., Sepúlveda-Vildósola A.C. (2019). Social Violence Increases the risk of suicidal ideation among undergraduate medical students. Arch. Med. Res..

[32] Valdez-Santiago R., Marín-Mendoza E., Torres-Falcón M. Análisis comparativo del marco legal en salud mental y suicidio en México. Salud Pública Méx..

[33] Borges G., Benjet C., Orozco R., Medina-Mora M.E., Menéndez D. (2017). Alcohol, cannabis and other drugs and subsequent suicide ideation and attempt among young Mexicans. J. Psychiatr. Res..

[34] Darvishi N., Farhadi M., Haghtalab T., Poorolajal J. (2015). Alcohol-related risk of suicidal ideation, suicide attempt, and completed suicide: A meta-analysis. PLoS ONE.

[35] Marschall-Lévesque S., Castellanos-Ryan N., Parent S., Renaud J., Vitaro F., Boivin M., Tremblay R., Séguin J. (2017). Victimization, Suicidal Ideation, and Alcohol Use from Age 13 to 15 Years: Support for the Self-Medication Model. J. Adolesc. Health.

[36] Herrera A., Benjet C., Méndez E., Casanova L., Medina-Mora M.E. (2017). How mental health interviews conducted alone, in the presence of an adult, a child or both affects adolescents’ reporting of psychological symptoms and risky behaviors. J. Youth Adolesc..

[37] González-Forteza C., Juárez-López C.E., Jiménez A., Montejo-León L., Rodríguez-Santisbón U.R., Wagner F.A. (2017). Suicide behavior and associated psychosocial factors among adolescents in Campeche, Mexico. Prev. Med..

[38] Cicchetti D., Rogosch F. (1996). Equifinality and multifinality in developmental psychopathology. Dev. Psychopathol..

[39] Valdez-Santiago R., Piña-Pozas M., Marín M.E., Martínez G.V., Chagoyán S.M., Gorbea-Portal S., Piña-Pozas M. (2021). Incremento de la Conducta Suicida Durante la Pandemia COVID-19: Revisión Rápida. References. Investigación y Metría de la Información Sobre Covid-19: Diversos Enfoques de la Pandemia.

[40] Pirkis J., John A., Shin S., DelPozo-Banos M., Arya V., Analuisa-Aguilar P., Appleby L., Arensman E., Bantjes J., Baran A. Suicide trends in the early months of the COVID-19 pandemic: An interrupted time-series analysis of preliminary data from 21 countries. Lancet Psychiatry.

